# Supplementary material for: MetaRibo-Seq measures translation in microbiomes
Source: Nat Commun. 2020 Jun 29;11:3268. doi: 10.1038/s41467-020-17081-z (PMC7324362; doi:10.1038/s41467-020-17081-z)
Supplement: Supplementary file 10 — Supplementary Data 7 [file 41467_2020_17081_MOESM10_ESM.zip › File2/Confidence_High_Taxonomy/324805_out.krona.html]

Javascript must be enabled to view this page.

members
magnitude
magnitudeUnassigned
count
unassigned
taxon
rank

324805\_out

14

superkingdom
2
10

10
phylum
1239

10
class
186801

10
186802
order

541000
family
10

genus
216851
10

species
853

SRS019808\_contig\_number\_3027SRS020233\_contig\_number\_33702SRS049896\_contig\_number\_19751SRS097920\_contig\_number\_21686SRS100021\_contig\_number\_8047SRS104912\_contig\_number\_17038SRS143181\_contig\_number\_contig-100\_3682.97763SRS148784\_contig\_number\_contig-100\_2962.140408SRS150029\_contig\_number\_4596SRS893378\_contig\_number\_12598
10


SRS049900\_contig\_number\_contig-100\_2302.86478SRS064757\_contig\_number\_contig-100\_23225.23226SRS143148\_contig\_number\_contig-100\_4246.46140SRS145497\_contig\_number\_contig-100\_11189.11189
4
